# Determination of major elements in igneous rocks using microwave plasma atomic emission spectrometry (MP-AES)

**DOI:** 10.1016/j.mex.2022.101793

**Published:** 2022-07-26

**Authors:** María Cecilia Geisenblosen, Pedro Oyhantçabal, Mariela Pistón

**Affiliations:** a Dirección Nacional de Minería y Geología, Ministerio de Industria Energía y Minería, Montevideo, Uruguay; b Graduate Program in Chemistry, Facultad de Química, Universidad de la República, Gral. Flores 2124, Montevideo, Uruguay; c Departamento de Geodinámica Interna, Instituto de Ciencias Geológicas, Facultad de Ciencias, Universidad de la República, Montevideo, Uruguay; d Grupo de Análisis de Elementos Traza y Desarrollo de Estrategias Simples para Preparación de Muestras (GATPREM), Analytical Chemistry, DEC, Facultad de Química, Universidad de la República, Gral. Flores 2124, Montevideo, Uruguay

**Keywords:** MP-AES, Igneous rocks, Mining, Geochemical Analysis, Major elements

## Abstract

An analytical method for sample preparation of igneous rocks and subsequent determination of Si, Na, K, Ca, Mg, Al, Fe, Mn, Ba, Sr, and Ti by means of microwave induced emission spectrometry (MP-AES) was developed and validated. The proposed sample preparation procedure avoids the use of perchloric acid and provided accurate results even for silicon determination using an acid digestion with hydrofluoric acid. The determination of major elements in rocks is required for the design of classification diagrams that provides relevant information for geochemical analysis.

• MP-AES showed to be an adequate technique to measure major and some trace elements that are relevant for classification of igneous rocks.
• This method is in better agreement with the principles of the Green Analytical Chemistry and constitutes a reliable alternative to classical analytical and tedious procedures used for geochemical analysis.
• The methodology was successfully applied to chemical classification of rocks from Valle Chico (Uruguay) using a Total Alkali-Silica Diagram (TAS).

## Graphical abstract

**Figure:**
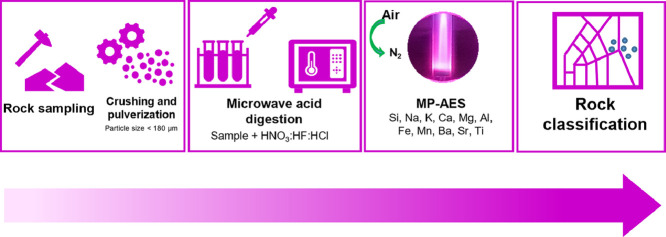

**SPECIFICATIONS TABLE**

**Table:** 

Subject Area;	Chemistry
More specific subject area;	*Analytical Chemistry*
Method name;	*Determination of Si, Na, K, Ca, Mg, Al, Fe, Mn, Ba, Sr, and Ti in igneous rocks by MP-AES*
Name and reference of original method;	*V. Balaram, V. Dharmendra, R. Parijat, T Craig, C.T. Kamalaa, M. Satyanarayanan, K. Prasenjit, K.S.V. Subramanyama, Arun Kumar Rajub, and A. Krishnaiah “Analysis of Geochemical Samples by Microwave Plasma-AES”, Atomic Spectroscopy, vol. 35 (2), pp. 65-78, 2014. DOI: 10.46770/AS.2014.02.003*
Resource availability;	*Not applicable*

*Method details

## Introduction

For decades, geologists have relied on geochemical data for the chemical classification of rocks and the comprehension of geological process using different types of diagrams. Conventionally, geochemical data used for these purposes are divided into major and trace elements. Major elements are those that predominate in any rock such as Si, Ti, Al, Fe, Mn, Mg, Ca, Na and K. On the other hand, trace elements are those present in rocks in concentrations of less than 0.1%. Some elements are considered trace elements in one type of rock and major elements in others. With the development of new techniques that allowed the determination of trace and ultra-trace elements and the increased application of these elements in geochemistry, it became essential to determine the chemical composition of rocks in a wide range of concentrations (from wt. % to µg g^−1^) [1].

Several atomic spectrometric techniques are available for geochemical analysis. The most widely used for trace element analysis are inductively coupled plasma mass spectrometry (ICP-MS) and instrumental neutral activation analysis (INAA). Since the introduction of commercial ICP-MS spectrometers, and due to its high sensitivity, multielement capability, selectivity, and precision it has been chosen as the gold technique for the determination of trace and ultratrace elements in rock samples [2], [3], [4], [5], [6], [7]. Despite the increased use of trace elements in geochemistry, major element determination continues to be essential and the most used techniques for its determinations are X-ray fluorescence (XRF) and inductively coupled plasma optical emission spectrometry (ICP OES) [8], [9], [10], [11].

Recently, microwave induced plasma optical emission spectrometry (MP-AES) was employed for major and some trace element determinations in geological samples with different sample preparation procedures obtaining good results [12,13]. One of the main advantages of the MP-AES technique is that the plasma uses nitrogen that can be obtained from air and that is the reason why they are promoted as an equipment that “runs on air”.

The acid dissolution of geological samples for their subsequent analysis by spectrometric techniques requires the use of hydrofluoric acid. Many authors reported difficulties with the determination of silicon after this treatment, associating it with losses due to the formation of volatile compounds or precipitation as fluoride complexes [12,14]. To avoid these difficulties the use of closed system digestion and HF complexation had been reported [13,14].

This analytical method introduces modifications in the sample preparation step that makes it simpler and in better agreement with the principles of the Green Analytical Chemistry than others reported that use perchloric acid or alkaline fusion. [12], [13], [14], [15], [16], [17]. This development contributes to obtain a reliable analytical method for rocks analysis, including silicon determination. Besides, an application that provides valuable geochemical data for rocks classification is described and discussed. This method is suitable for the chemical characterization of rocks and of interest in mineral exploration.

### Chemicals and materials

The standard reference solutions used for calibration were prepared in 2% (w/w) nitric acid by adequate dilution of the ICP Multi-element Standard Solution IV (Merck, Darmstadt, Germany) for Al, Ba, Ca, Fe, K, Mg, Mn, Na and Sr, in the case of Si a Certipur Silicon Standard Solution (Merck, Darmstadt, Germany) was employed and for Ti a Pure Standard (Perkin Elmer, USA) was used.

All the solutions were prepared with ultrapure water obtained from a Milli-QTM system (18 MΩ cm, Millipore, Bedford, MA, USA).

For sample preparation hydrofluoric acid (48% w/w, reagent A.C.S., Carlo Erba, Italy) was distilled using a PFA sub-boiling distillation system (Sub-clean, Millestone, USA). Nitric acid (65% w/w, reagent A.C.S., Merck, Darmstadt, Germany) and hydrochloric acid (37% w/w, reagent A.C.S., Merck, Darmstadt, Germany) were distilled in a quartz sub-boiling distillation system (DuoPur, Milestone, Sorisole, Italy). Boric acid, analytical grade (Merck, Darmstadt, Germany) was used for fluoride complexation after digestion of the samples. For trueness evaluation of the developed method a Certified Reference Material (CRM) of ryholite RGM-2 (United State Geological Survey, USA), was analyzed following the same procedure used for the collected samples, this material of igneous volcanic rock was selected considering that it is a very similar matrix to the samples.

### Analytical determinations

Analytical determinations of Si, Na, K, Ca, Mg, Al, Fe, Mn, Ba, Sr, and Ti were performed using a microwave-induced plasma optical emission spectrometer 4210 (MP-AES Agilent Technologies, Santa Clara, USA) equipped with an autosampler model SPS 4 (Agilent Technologies, Santa Clara, USA). Sample introduction was by means of an inert One Neb nebulizer with a double-pass glass cyclonic spray chamber system, and a standard torch. The spectrometer used an online nitrogen generator model 4107 (Agilent Technologies, Santa Clara, USA), which takes in air from the environment through an air compressor model KK70 TA-200 K (Dürr Technik, Bietigheim-Bissingen, Germany).

Operating conditions and instrumental parameters are presented in Table 1. Before the measurements, the viewing position and nebulizer flow were optimized for each element using a 2 mg L^−1^ standard solution containing all the studied elements. The optimization is performed by the commercial software of the instrument. The optimized conditions are shown in Table 2 where the selected wavelengths for each element are also listed. Sample composition using the autosampler module is about 3 mL for a triplicate lecture.

**Table 1:** Operating parameters used in Agilent 7800 Quadrupole ICP-MS.

Instrument parameter	Operating condition
Microwave frequency (MHz)	2450
Applied plasma power (kW)	1.0
Stabilization time (s)	15
Background correction	Auto
Reading time (s)	3
**Sample introduction system**	
Nebulizer	OneNeb (Series 2)
Spray chamber	Single-pass glass cyclonic
Torch	easy-fit (1.8 mm id)
Microwave frequency (MHz)	2450
Applied plasma power (kW)	1.0
Stabilization time (s)	15
Background correction	Auto

**Table 2:** MP-AES measurement conditions.

Element	Wavelength (nm)	Viewing position	Nebulizer gas flow (L min^−1^)
Si	251.611	0	0.45
Na	588.995	-40	0.95
Mg	285.213	10	0.75
Al	396.152	0	0.95
K	766.491	0	1.0
Ca	393.366	20	0.70
Ti	323.452	10	0.60
Mn	403.076	10	1.0
Fe	371.993	20	0.80
Sr	407.771	20	0.75
Ba	455.403	10	0.90

## Sampling and sample preparation

### Samples

For the development of this method rock samples were taken from the Valle Chico igneous Complex located in Southeastern Uruguay. This complex covers approximately 250 km^2^ with an approximate NE–SW-oriented elliptical shape. Muzio [18] and Lustrino et al. [19] studied this massif and presented a geochemical characterization. However, due to the extension of the massif, there are some areas of the complex not fully characterized. Therefore, samples were selected from this location to apply the proposed analytical method.

**Fig. 1:**
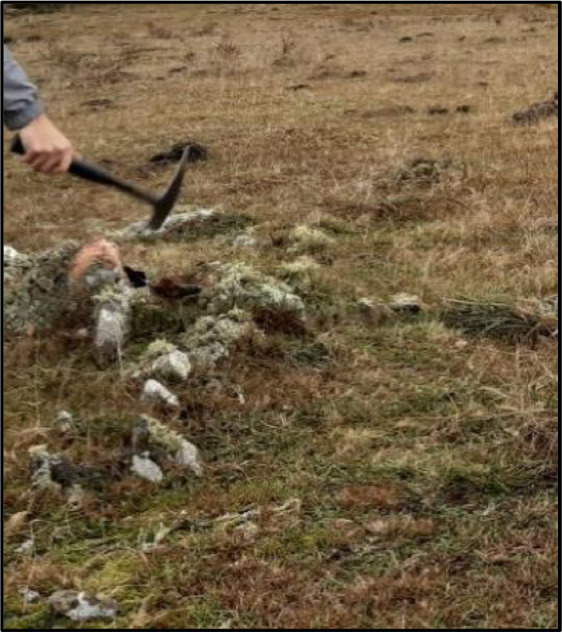
Rock sampling.

**Fig. 2:**
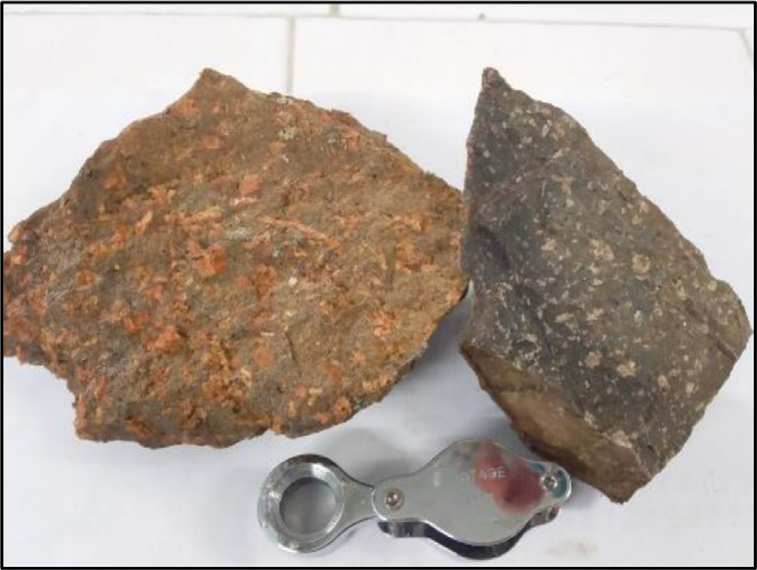
Samples from Valle Chico dykes.

Seventeen samples of volcanic igneous rocks were selected for method validation and application, these samples were taken from a dyke swarm in the Valle Chico massif using a steel rock pick to access fragments of sample not exposed to weather alteration. Figs. 1 and 2 illustrate the sampling process.

Thin section petrography of the samples did not show any significant alteration in the primary rock-forming minerals and thus the samples are considered not altered.

About 2 kg of rock material was collected for each sample and was dried in an oven (Memmert, Büchenbach, Germany) at 40°C for 48 h. Once dried, they were crushed using a Denver N°2 jaw crusher to reduce the particle size to less than 6 cm and after that using a Fritsch Pulverisette 1 (Fritsch, Idar-Oberstein, Germany) jaw crusher with tungsten carbide jaws, the particle size was reduced to less than 1 cm. Following, samples were divided using a sample splitter and approximately 200 g were pulverized with an agate ring mill, Jurgens Siebtechnik TS 100A (Siebtechnik, Mülheim, Germany) for 15 min to obtain a final particle size of an 85% of distribution less than 125 μm.

Acid digestion was carried out using a Multiwave 3000 (Anton Paar, Graz, Austria) microwave system, equipped with an HF100 rotor with 16 PTFE-TFM 100 mL vessels.

Sample weight for the microwave assisted digestions is a very important variable since homogeneity should be guaranteed and it defines the detection limits. Using the minimum acid volume recommended by microwave manufacturers when common vessels of 100 mL are used (6 mL), different sample weights were evaluated to obtain an efficient total digestion. In this sense, 0.05; 0.10; 0.15; 0.20 and 0.25 g of the powdered sample were digested using the same reagents and microwave temperature program [10], [11],20]. The solutions obtained after the acid digestion for up to 0.20 g of sample were clear and this is a reasonable mass frequently recommended for the use of certified reference materials. Thus, for samples and reference material, 0.2 g were accurately weighed and transferred to the microwave vessels. Additionally, 3.0 mL of HNO_3_, 1.0 mL of HCl and 3.0 mL of HF were added to the vessels. The samples were digested for 60 min at 1400 W (5 min ramp + 60 min hold time). A second step for complexation of the remaining HF was carried out by adding 18.0 mL of H_3_BO_3_ 5% w/w and then starting a program of heating in the microwave for 15 min at 1400 W (5 min ramp + 15 min hold time). The resulting solutions were diluted up to 50.00 mL with ultrapure water (250-fold dilution) and further dilutions were made when needed. Analytical blanks and the CRM were digested using the same procedure. All samples and CRM were digested in duplicate (except for validation where more replicates were performed).

After completing the sample preparation procedure, samples, standards, and the CRM were analyzed by means of MP-AES under the operative conditions described before. Under these conditions the total dissolved solids (TDS) were appropriate for the technique (less than 2%).

## Method validation

The method validation was performed according to the recommendations of Eurachem Guide [21]. The figures of merit studied were dynamic range, limit of detection (LOD), limit of quantification (LOQ), precision and trueness. Precision and trueness were evaluated using the CRM RGM-2. Trueness was evaluated comparing the obtained values with those from the certificate (USGS) and expressed as the mean recovery (*n* = 5) ± standard deviation (R%) and precision as relative standard deviation (RSD %, *n* = 6).

The calibration curves were prepared in 2% (w/w) HNO_3_ since no significant difference was obtained in the slope of the calibration curves when they were tested using the same reagents of the digestion process (less than 2% of variation), this is a great practical advantage that saves time and makes the procedure cheaper.

The LOD and LOQ were estimated for each element as 3 σ/b and 10 σ/b, respectively, where σ was the standard deviation of 10 replicates (*n* = 10) of the digestion reagent blanks fortified with a standard solution at a low concentration (equivalent to 6.25 µg g^−1^ in solid sample) and b was the slope of the calibration curve. The addition of the analytes to the blanks for these determinations was required since the single blank signal was erratic due to the influence of boric acid in the matrix. For a general routine analysis of this kind of samples, fortification of the blanks to estimate LOD and LOQ values is recommended at a level of 5 to 10 µg g^−1^ to avoid mistakes or high uncertainly in the estimation of this figure of merit. In general, for major elements, these values are not critical, but they are important to highlight the capabilities of the MP-AES technique.

Silicon LOD and LOQ were not informed since it is normally present in very high concentrations in this kind of samples.

The validation was successful yielding the figures of merit shown in Table 3.

**Table 3:** Figures of merit.

Element	LOD (3σ, *n* = 10) (μg g^−1^)	LOQ (10σ, *n* = 10) (μg g^−1^)	Dynamic range (mgL^−1^)	Correlation coefficient R^2^	Precision (RSD %, *n* = 6)
Si	-	-	0.05–10	0.9994	3.3
Na	110	320	0.05–10	0.9998	1.1
Mg	1.0	2.0	0.5–5.0	1.0000	4.2
Al	100	300	0.05–10	0.9999	6.6
K	140	400	0.05–5.0	0.9998	1.3
Ca	4.0	13	0.5–10	0.9999	2.5
Ti	6.0	19	0.5–10	0.9999	1.9
Mn	1.0	4.0	0.5–10	0.9999	5.0
Fe	70	210	0.05–10	0.9999	3.8
Sr	0.3	0.8	0.05–1.0	0.9995	10
Ba	1.0	4.0	0.05–10	1.0000	8.4

Good linearity was observed in the studied dynamic ranges, selected according to the application, with determination coefficients (R^2^) greater than 0.999. Individual residuals were also studied, and its random distribution was verified.

Precision expressed as RSD% of the analysis of 6 replicates (*n* = 6) of the CRM was less than 10% for all the studied analytes, this was considered suitable for this application.

LODs and LOQs obtained were very adequate for the objectives of this study since these elements normally occur in high concentrations in these kinds of samples. Besides, these limits are very good compared with the ones reported for XRF and ICP OES in previous works [8,12].

For trueness evaluation, a Student's *t*-test was performed to compare the obtained values with the ones provided by the CRM supplier [22]. Results are presented in Table 4. Experimental *t*-values were below the t-theoretical indicating that, at the 95% confidence level, the concentrations did not differ significantly from the certified or informed value. As the R% turned out to be statistically equal to 100% for all the studied elements, with an adequate precision this method resulted to be accurate.

**Table 4:** Trueness evaluation.

Element	Si	Na	Mg	Al	K	Ca	Ti	Mn	Fe	Sr	Ba
R (%) *	102.0	100.2	99.0	93.3	98.6	95.8	100.8	99.2	97.2	108.4	102.8
***t- experimental***	1.35	- 0.09	- 0.50	- 2.53	-0.43	-2.54	0.16	- 1.63	- 0.92	0.96	0.83

*mean recovery (%) [Obtained value/certified value] x100; *t- theoretical* = 2.78; *n* = 5 [21].

The obtained figures of merit demonstrate that the developed method is reliable for the determination of these analytes in this matrix.

### Application

The seventeen collected samples of igneous rocks were treated using the sample preparation procedure described above.

For all of them a clear solution was obtained after the acid treatment and complexation. An adequate dilution with ultrapure water was performed before analysis by MP-AES (TDS < 2%), and the results are presented in Table 5. For Sr and Ba the concentrations found were in several samples at trace levels, which demonstrates that this technique can be useful also to determine these elements in the order of ppm.

**Table 5:** Major element composition of igneous rocks from Valle Chico (Uruguay).

Sample code	SiO_2_ wt. %	Al_2_O_3_ wt. %	Fe_2_O_3_ wt. %	Na_2_O wt. %	K_2_O wt. %	CaO wt. %	MgO wt. %	MnO wt. %	TiO_2_ wt. %	Ba ppm	Sr ppm
VHC140	76 ± 2	11.20.7	6.2 ± 0.2	4.17 ± 0.05	4.27 ± 0.06	0.31 ± 0.01	0.10 ± 0.01	0.14 ± 0.01	0.36 ± 0.01	125± 10	8 ± 1
VHC142B	67 ± 2	15.9 ± 1.0	7.3 ± 0.3	3.41 ± 0.04	5.06 ± 0.07	0.18 ± 0.01	0.27 ± 0.01	0.06 ± 0.01	0.66 ± 0.01	2657 ± 223	113 ± 11
VHC144	71 ± 2	12.6 ± 0.8	10.3 ± 0.4	3.86 ± 0.04	4.21 ± 0.05	0.28 ± 0.01	0.08 ± 0.01	0.21 ± 0.01	0.53 ± 0.01	72 ± 6	ND
VHC145	76 ± 3	11.2 ± 0.7	4.0 ± 0.2	4.20 ± 0.05	2.48 ± 0.03	0.07 ± 0.01	0.10 ± 0.01	0.03 ± 0.01	0.19 ± 0.01	77 ± 6	13 ± 1
VCH149	61 ± 2	15.3 ± 1.0	6.2 ± 0.2	5.60 ± 0.06	5.05 ± 0.07	0.66 ± 0.02	0.16 ± 0.01	0.17 ± 0.01	0.51 ± 0.01	120 ± 10	21 ± 2
VCH151	68 ± 2	11.1 ± 0.7	5.7 ± 0.2	4.39 ± 0.05	4.24 ± 0.05	0.52 ± 0.01	0.07 ± 0.01	0.13 ± 0.01	0.29 ± 0.01	107 ± 9	25 ± 3
VCH158	74 ± 2	9.7 ± 0.6	6.1 ± 0.2	4.21 ± 0.05	4.50 ± 0.06	0.25 ± 0.01	0.06 ± 0.01	0.12 ± 0.01	0.32 ± 0.01	49 ± 4	4.5 ± 0.5
VCH160	64 ± 2	15.6 ± 1.0	6.0 ± 0.2	6.09 ± 0.07	4.80 ± 0.06	0.61 ± 0.02	0.23 ± 0.01	0.14 ± 0.01	0.43 ± 0.01	429 ± 36	61 ± 6
VCH162	71 ± 2	9.4 ± 0.6	7.8 ± 0.3	3.95 ± 0.04	4.40 ± 0.06	0.18 ± 0.01	0.04 ± 0.01	0.16 ± 0.01	0.34 ± 0.01	103 ± 9	5.6 ± 0.6
VCH165	73 ± 2	14.4 ± 1.0	6.7 ± 0.3	4.69 ± 0.05	5.22 ± 0.07	0.23 ± 0.01	0.12 ± 0.01	0.13 ± 0.01	0.55 ± 0.01	324 ± 27	19 ± 2
VCH201	70 ± 2	13.1 ± 0.9	4.7 ± 0.2	3.53 ± 0.04	4.59 ± 0.06	0.26 ± 0.01	0.10 ± 0.01	0.08 ± 0.01	0.29 ± 0.01	149 ± 13	15 ± 2
VCH202	65 ± 2	15.0 ± 1.0	5.9 ± 0.2	4.94 ± 0.05	4.62 ± 0.06	0.38 ± 0.01	0.22 ± 0.01	0.15 ± 0.01	0.46 ± 0.01	187 ± 16	22 ± 2
VCH212	74 ± 2	11.4 ± 0.8	2.2 ± 0.1	1.68 ± 0.02	4.96 ± 0.07	0.14 ± 0.01	0.10 ± 0.01	0.03 ± 0.01	0.21 ± 0.01	1068 ± 90	52 ± 5
VCH215	72 ± 2	12.4 ± 0.8	2.7 ± 0.1	1.09 ± 0.01	5.48 ± 0.07	0.11 ± 0.01	0.17 ± 0.01	0.02 ± 0.01	0.23 ± 0.01	653 ± 55	30 ± 3
VCH216	73 ± 2	12.3 ± 0.8	2.4 ± 0.1	1.95 ± 0.02	5.61 ± 0.07	0.13 ± 0.01	0.19 ± 0.01	0.02 ± 0.01	0.21 ± 0.01	556 ± 47	26 ± 2
VCH220	69 ± 2	12.7 ± 0.8	4.0 ± 0.1	1.34 ± 0.01	4.88 ± 0.06	0.13 ± 0.01	0.18 ± 0.01	0.03 ± 0.01	0.33 ± 0.01	473 ± 40	21 ± 2
VCH222	65 ± 2	13.8 ± 0.9	4.7 ± 0.2	2.70 ± 0.03	5.57 ± 0.07	0.36 ± 0.01	0.16 ± 0.01	0.07 ± 0.01	0.33 ± 0.01	756 ± 64	68 ± 7

Results expressed as mean (*n* = 2) ± standard deviation. ND: not detected

Despite the constant develop of new classification diagrams adequate even for altered samples [23], the major elements measured in this investigation only allowed the use of the TAS and R1-R2 diagrams as an application of the proposed analytical method.

**Fig. 3:**
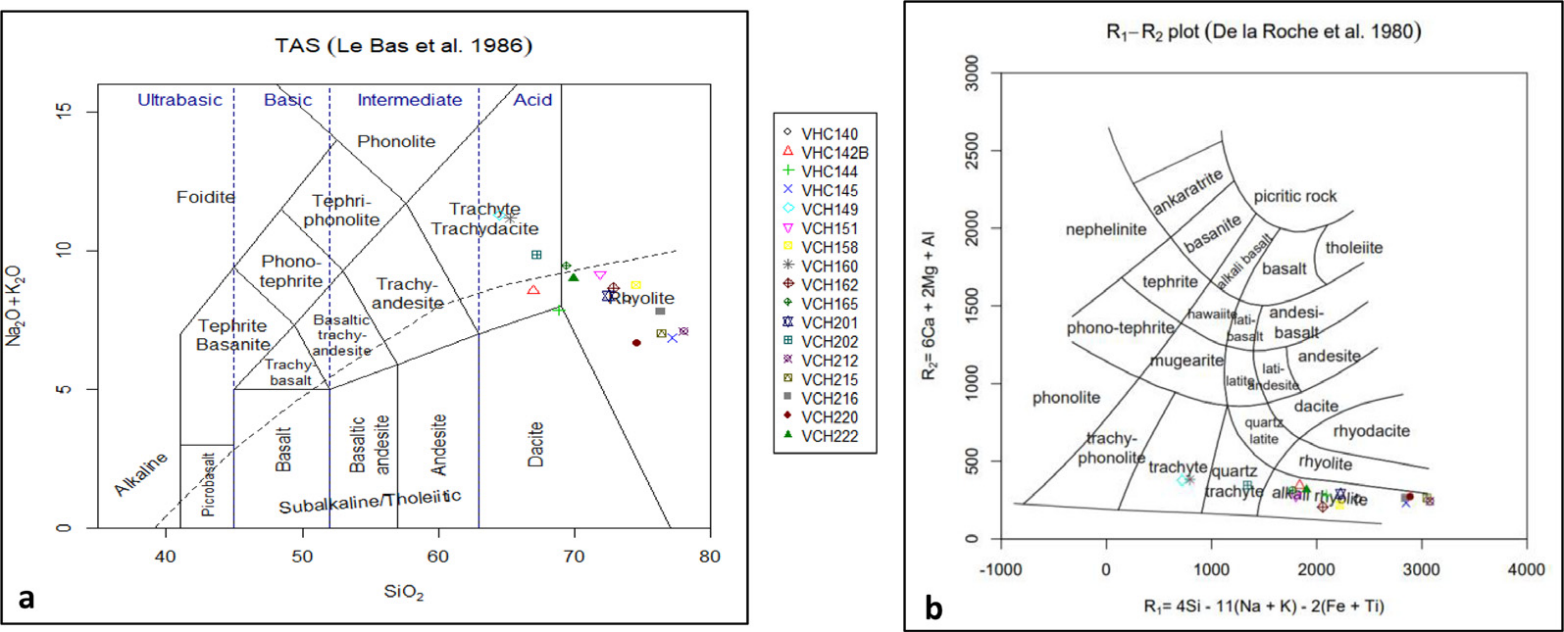
(a) Total Alkali-Silica diagram (TAS, [25]); (b) R1-R2 diagram [28].

The Total Alkali-Silica Diagram (TAS) was first presented by Le Maitre et al. In 1984 [24], and later modified by Le Bas et al. in 1986 [25]. In this diagram, recommended by the Subcommission on the Systematics of igneous Rocks of the International Union of Geological Sciences [26], samples are classified according to their relationship between the content of SiO_2_ wt. % and the sum of NaO_2_ wt. % and KO_2_ wt. %. In Fig. 3, the results of the analyzed samples (Table 5) were plotted on the TAS diagram using the GeoChemical Data ToolKIT (GCDKit) software [27]. According to this all the samples were classified as trachyte/trachydacite and rhyolite. This classification agreed with the results published by Muzio et al. in 2002 where only a few dike samples of Uruguay were analyzed [18]. For comparison, the classification on the R1-R2 diagram [28] is shown in Fig. 3b.

Thus, our new data provide more exhaustive information for geochemical classification of rocks of this massif based on Total Alkali-Silica and the R1-R2 diagrams.

## Conclusion

MP-AES, that basically runs on air, resulted an economical and sustainable alternative to the use of more expensive techniques that require argon for this application. The validated method can be performed in laboratories to obtain relevant and reliable information about major (including silicon) and some trace element composition of rocks to perform geochemical studies.

## Declaration of Competing Interests

The authors declare that there is no conflict of interest regarding the publication of this article.

## Data Availability

data will be available in the PhD thesis of the first author on the public repository of the Universidad de la Republica https://www.colibri.udelar.edu.uy/jspui/handle/20.500.12008/38.

## References

[1] Rollinson H.R. (1993).

[2] Pinto F.G., Junior R.E., Saint´Pierre T.D. (2012). Sample preparation for determination of rare earth elements in geological samples by ICP-MS: a critical review. Anal. Lett..

[3] Fedyunina N.N., Seregina I.F., Bolshov M.A., Okina O.I., Lyapunov S.M. (2012). Investigation of the efficiency of the sample pretreatment stage for the determination of the rare earth elements in rock samples by inductively coupled plasma mass spectrometry technique. Anal. Chim. Acta.

[4] Okina O.I., Lyapunov S.M., Dubensky A.S. (2018). Influence of sample treatment after bomb digestion on determination of trace elements in rock samples by ICP-MS. Microchem. J..

[5] Zhang W., Qi L., Hu Z., Zheng C., Liu Y., Chen H., Gao S., Hu S. (2015). An investigation of digestion methods for trace elements in bauxite and their determination in ten bauxite reference materials using inductively coupled plasma-mass spectrometry. Geostand. Geoanal. Res..

[6] Chauvel C., Bureau S., Poggi C. (2010). Comprehensive chemical and isotopic analyses of basalt and sediment reference materials. Geostand. Geoanal. Res..

[7] Hai N.C., Dien N.N., Tan V.H., Anh T.T., Son P.N., Thang H.H. (2019). Determination of elemental concentrations in biological and geological samples using PGNAA facility at the Dalat research reactor. J. Radioanal. Nucl. Chem..

[8] Ogasawara M., Mikoshiba M., Geshi N., Shimoda G., Ishizuka Y. (2018). Optimization of analytical conditions for major element analysis of geological samples with XRF using glass beads. Bull. Geol. Surv. Jpn..

[9] Verma S.P., Rosales-Rivera M., Rivera-Gómezd M.A., Vermael S.K. (2019). Comparison of matrix-effect corrections for ordinary and uncertainty weighted linear regressions and determination of major element mean concentrations and total uncertainties of sixty-two international geochemical reference materials from wavelength-dispersive X-ray fluorescence spectrometry. Spectrochim. Acta Part B.

[10] Murray R.W., Miller D.J., Kryc K.A. (2000). Analysis of major and trace elements in rocks, sediments, and interstitial waters by inductively coupled plasma–atomic emission spectrometry (ICP-AES). Ocean Drilling Program ODP, Technical Note.

[11] Briggs P.H. (2001). Analytical Methods for Chemical Analysis of Geologic and Other Materials.

[12] Balaram V., Dharmendra V., Parijat R., Craig T., Kamalaa C.T., Satyanarayanan M., Prasenjit K., Subramanyama K.S.V., Raju A.K., Abburi K. (2014). Analysis of geochemical samples by microwave plasma-AES. At. Spectrosc..

[13] Niedzielski P., Kozak L., Wachelka M., Jakubowski K., Wybieralska J. (2015). The microwave induced plasma with optical emission spectrometry (MIP–OES) in 23 elements determination in geological samples. Talanta.

[14] Santos M.A., Silva A.B.S., Machado R.C., Dias E.A., Barros J.A.V.A., Nogueira A.R.A. (2020). Silicon determination by microwave-induced plasma optical emission spectrometry: Considerations and strategies for the use of tetrafluorboric acid and sodium hydroxide in sample preparation procedures. Spectrochim. Acta Part B.

[15] Whitty-Léveillé L., Turgeon K., Bazin C., Lariviére D. (2017). A comparative study of sample solution techniques and plasma-based instruments for the precise and accurate quantification of REEs in mineral matrices. Anal. Chim. Acta.

[16] Balaram V. (2020). Microwave plasma atomic emission spectrometry (MP-AES) and its applications – a critical review. Microchem. J..

[17] Williams C.B., Amais R.S., Fontoura B.M., Jones B.T., Nobrega J.A., Donati G.L. (2019). Recent developments in microwave-induced plasma optical emission spectrometry and applications of a commercial Hammer-cavity instrument. Trends Anal. Chem..

[18] Muzio R. (2002). Petrological and geochemical evolution of the Valle Chico Alkaline Massif, Southeastern Uruguay. Int. Geol. Rev..

[19] Lustrino M., Melluso T.L., Brotzu P., Gomes C.B., Morbidellia L., Muzio R., Ruberti E., Tassinari C.C.G. (2005). Petrogenesis of the early Cretaceous Valle Chico igneous complex (SE Uruguay): relationships with Parana–Etendeka magmatism. Lithos.

[20] Totland M., Jarvis I., Jarvis K.E. (1992). An assessment of dissolution techniques for the analysis of geological samples by plasma spectrometry. Chem. Geol..

[21] Eurachem Guide: The fitness for purpose of analytical methods – a laboratory guide to method validation and related topics (2014) www.eurachem.org. Accessed December 2020

[22] Miller J.N., Miller J.C. (2010).

[23] Verma SP. (2020).

[24] Le Maitre R.W., Streckeisen A., Zanettin B., Le Bas M.J., Bonin B., Bateman P., Bellieni G., Dudek A., Efremova S., Keller J., Lameyre J., Sabine P.A., Schmid R., Sørensen H., Woolley A.R. (2002). Igneous Rocks: A Classification and Glossary of Terms.

[25] Le Bas M.J., Le Maitre R.W., Streckeisen A., Zanettin B. (1986). A chemical classification of volcanic rocks based on the total Alkali-Silica diagram. J. Petrol..

[26] Le Bas M.J., Streckeisen A. (1991). The IUGS systematics of igneous rocks. J. Geol. Soc..

[27] Janousek V., Farrow C.M., Erban V. (2006). Interpretation of whole-rock geochemical data in igneous geochemistry: introducing geochemical data toolkit (GCDkit). J. Petrol..

[28] De La Roche H., Leterrier J., Grandclaude P., Marchal M. (1980). A classification of volcanic and plutonic rocks using R1R2-diagram and major-element analyses - its relationships with current nomenclature. Chem. Geol..

